# Development and evaluation of a novel fast broad-range 16S ribosomal DNA PCR and sequencing assay for diagnosis of bacterial infective endocarditis: multi-year experience in a large Canadian healthcare zone and a literature review

**DOI:** 10.1186/s12879-016-1476-4

**Published:** 2016-04-12

**Authors:** Robert J. H. Miller, Barbara Chow, Dylan Pillai, Deirdre Church

**Affiliations:** Department of Cardiac Sciences, University of Calgary, Calgary, Alberta T2N 2T9 Canada; Calgary Laboratory Services, Calgary, Alberta T2L 2K8 Canada; Departments of Pathology & Laboratory Medicine and Medicine, University of Calgary, and Calgary Laboratory Services, 9-3535 Research Rd. NW, Calgary, Alberta T2L 2K8 Canada

**Keywords:** Endocarditis, Broad-range 16S rDNA PCR, Bacterial pathogen, Molecular diagnosis

## Abstract

**Background:**

The study aimed to explore the sensitivity and specificity of a novel fast 16S rDNA PCR and sequencing assay for the improved diagnosis of infective endocarditis (IE) in patients with suspected native or prosthetic heart valve (HV) infection over a multi-year period at our cardiovascular center.

**Methods:**

Sixty-eight patients were prospectively enrolled who underwent HV replacement for suspected or confirmed IE between February 1, 2009 and September 1, 2014. Patient demographics, medical co-morbidities, Duke’s criteria, culture results, and antibiotic therapy were collected by detailed chart reviews. Dual-priming oligonucleotide primers targeted to 500 bps of the V1-V3 region of the 16S rRNA gene were used to perform fast broad-range 16S rDNA PCR and Sanger sequencing on ribosomal DNA extracted from HV tissues. The performance/diagnostic efficiency of the molecular test was evaluated against blood cultures and Gram stain and culture of HV tissue in patients’ with definite IE according to Duke’s criteria.

**Results:**

Fifty patients (73.5 %) had definite IE and another 8 (11.8 %) had possible IE according to Duke’s criteria. Cardiac surgery was delayed an average of 15.4 days from the time of the patient’s last positive blood culture, and appropriate antibiotic therapy was given in the pre-operative period. While 44/50 (88 %) patients had a positive blood culture, HV tissue culture was only positive in 23 (46 %) of them. Molecular testing of all HV tissues had sensitivity, specificity, NPV and PPV of 92, 77.8, 77.8 and 92 % compared to 44, 100, 39.1 and 100 % respectively for culture for diagnosis of definite IE. For prosthetic HV tissue, 16S rDNA PCR had sensitivity of 93 % and specificity of 83 % compared to 35 and 100 % respectively for culture. A literature review showed that the diagnostic accuracy of our novel fast broad-range 16S rDNA PCR assay was similar or better than that of previously published studies.

**Conclusions:**

This novel fast broad-range 16S rDNA PCR/sequencing test had superior sensitivity compared to tissue Gram stain and culture for identifying underlying bacterial pathogen in both native and prosthetic valve endocarditis.

## Background

Infective endocarditis (IE) has a high morbidity and mortality, often requiring treatment in an intensive care unit [1, 2]. The Duke criteria are used to diagnose IE and may help determine whether cardiovascular surgery may be required [3], with almost half (44.9 %) of patients ultimately requiring valve surgery [2, 4]. Microbiologic diagnosis in IE, which is a cornerstone of the Duke criteria, has classically relied on traditional culture results for isolation of a pathogen(s) from blood or affected heart valve tissue. However, blood culture results are negative in 9–13 % of patients and all cultures are negative in 5 % of patients [2, 5]. Negative culture results are even more prevalent in patients with atypical organisms and prosthetic valve IE due to difficulties in selecting appropriate tissue [6–10]. The rates of culture-negative IE with atypical or difficult to culture bacteria have recently increased [2]. Identification of the causative pathogen is important for targeting antimicrobial therapy as well as determining prognosis.

Broad-range sequencing of bacterial 16S ribosomal DNA represents an alternative approach for establishing the underlying organism in IE. Since broad-range sequencing does not rely on culturing the underlying organism, it may be particularly useful for identifying pathogens that aren’t readily cultured with current methods [7, 11]. Evidence supporting the use of broad-range 16S rDNA PCR/sequencing for molecular diagnosis in some cases series show that heart valve PCR may improve microbiological diagnosis of IE in up to 20 % of patients. Some authors have even suggested it be made part of Duke’s diagnostic criteria [12]. The routine molecular diagnosis of IE has so far been evaluated in a small number of patients, primarily in European centers [9, 13–20]. Because broad-range PCR assays are prone to contamination [21], and primers within the 16S rRNA gene may cross-react with human DNA in clinical samples, we developed a novel, fast broad-range 16S rDNA PCR using unique dual-priming oligonucleotide (DPO) primer to try and increase the sensitivity and specificity of molecular diagnosis of IE [22]. The performance of our novel 16S rDNA PCR/sequencing test was then validated by comparing it with the results of blood culture and Gram stain and culture of excision heart valve tissue in patients with and without a clinical diagnosis of IE. A literature review was also done in order to compare our results to other similar published diagnostic accuracy studies of other user-developed PCR assays [9, 13–19, 23–30], and recently published evaluations of commercial PCR tests [31, 32].

## Methods

### Study setting and patients

The Calgary Zone is one of the largest integrated healthcare jurisdictions in Canada, which provides patient care services to an urban and rural population of ~1.5 million people. From February 1, 2009 to September 1, 2014, 68 patients with suspected IE underwent valve replacement surgery at the Division of Cardiovascular Surgery, Foothills Medical Center, Calgary Zone, Alberta Health Services, Canada. The cardiovascular service is a tertiary referral centre in the western part of Canada that primarily provides patient care to Southern Alberta, but is also referred patients from other adjacent provinces.

Patients were retrospectively reviewed after surgery using a standardised data collection sheet. Clinical features for all patients were collected, including: age, sex, type of valve tissue, location of valve, all blood culture results, tissue culture results, peak white blood cell count (WBC), peak erythrocyte sedimentation rate (ESR), and peak C-reactive protein (CRP). Additionally history collected included; previous IE, prosthetic valve, intravenous drug use, and other predisposing factors. Antibiotic use in the peri-operative period and in relation to culture results was also recorded. We classified patients as definite, possible, or rejected diagnosis of IE based on the modified Duke’s criteria [3].

### Laboratory setting and samples

Microbiology testing was performed by the Division of Microbiology, Calgary Laboratory Services (CLS). CLS is a large regional centralized laboratory that performs diagnostic testing for the entire Calgary Zone, including all ambulatory, hospitalized and long-term care patients. CLS Microbiology performs complex testing on >1 million individual patient samples per annum.

Cardiac surgeons collected heart valves under sterile conditions during each operative procedure. Heart valves were immediately placed into a sterile container and promptly transported to the Calgary Laboratory Services (CLS) microbiology laboratory within 2 h after excision. Heart valve tissue was stored at −80 to −86 °C and batched for DNA extraction.

### Blood and heart valve cultures

All patients had blood cultures performed prior to the prescription of antibiotics. Two separate sets of blood cultures [BacT/Alert FA (Aerobic) and FN (Anaerobic)] are routinely drawn when adult blood cultures are ordered. Blood cultures were immediately transported to CLS and placed into the BacT/Alert system, and continuously monitored for up to 4-days for growth. Blood culture bottles that flagged positive in the instrument were immediately removed and an aliquot removed, pelleted and plated onto routine media including a Columbia blood agar (BA), chocolate agar (CHOC), MaConkey (MAC) agar, and Brucella blood agar (BBA) for culture of both aerobic and anaerobic bacteria. Isolates were identified using a combination of rapid phenotypic methods and MALDI-TOF (Vitek MS MALDI-TOF, bioMérieux, Laval Que.).

Native heart valve tissue was aseptically disrupted in a tissue grinder in 0.5 mL brain heart infusion (BHI) broth. Prosthetic valves were covered in 2.5 mL BHI and vortexed for 30 s. before and after sonication for 5 min. After sonication, the BHI tube was centrifuged at 3000 × *g* relative centrifugal force for 15 min. and the supernatant was discarded. The 0.5 mL sediment was then re-suspended by vortexing for 15 s. Aliquots (100 μL) of the prepared heart valve tissue sediments were immediately inoculated into a BacT/Alert FA (Aerobic) and FN (Anaerobic) blood culture bottle and placed into the BacT/Alert (bioMérieux, Saint-Lauren, QC) cabinet for continuous incubation and monitor for up to 7 days. A 20–30 μL drop of the sediment was planted onto BA, CHOC and BBA agars for culture of both aerobic and anaerobic bacteria. BA and CHOC plates were routinely incubated in oxygen at 35 °C for up to 7 days, while the BBA plate underwent anaerobic incubation in an Anoxamat cabinet (Mart Microbiology, Inc., Drachten, Netherlands) for the same period of time. Blood culture bottles were terminally sub-cultured onto all of the above media and the plates incubated for a further 7 days. Fungal culture was also routinely performed by inoculation of inhibitory mould agar (IMA) and brain heart infusion agar (BHIA), and fungal plates were incubated in 0_2_ at 30 °C for up to 6 weeks. A separate piece of heart valve tissue as well as the tissue sediment was stored frozen at −86 °C for molecular testing.

### Molecular methods

Each heart valve tissue was minced manually with sterile scalpels before being placed into a sterile 1.5 mL micro-centrifuge tube. DNA was extracted using the QIAamp DNA Mini Kit protocol for tissues (QIAGEN, Hilden, Germany). Briefly, the tissue was re-suspended in 180 μL of digestion Buffer ATL (50 mM Tris HCl, 1 mM EDTA, 0.5 % SDS, pH 8.5) and 20 μL of 20 mg/mL Proteinase K solution (QIAGEN). The samples were then briefly vortexed and incubated at 56 °C, in a 1400 rpm Eppendorf thermomixer for a minimum of 2 h. DNA in the proteolytic digest was further purified according to the manufacturer’s instructions. DNA was eluted in 150 μL of Buffer AE. DNA eluate was stored at −20 °C until use.

Broad-range 16S rDNA PCR was performed using dual priming oligonucleotide (DPO) primers previously described by Kommedal et al. [33] including a forward primer [16SDPO_F:AGAgTTTgATCMTGGCTCA-I-I-I-I-I-AACGCT (M = A/C; I = deoxyinosine; lower case letter denotes a locked nucleic acid)], and a reverse primer [16SDPO_R:CGCGGCTGCTGGCA-I-I-I-A-I-TTRGC (R = A/G; I = deoxyinosine)] purchased from Exiqon (Worburn, MA). PCR was set up in a 25 μL reaction volume with 5 μL of template DNA and 0.5 μL of each primer in a master mix that included 13 μL DNA-free water (Molzym, Bremen, Germany), 2.5 μL of 10× buffer (Minerva BioLabs, Berlin, Germany), 2.5 μL of 2 mM each dNTP mix prepared in-house, and 0.625 μL of 2U/μL Eub polymerase (Minerva BioLabs). Standard Fast PCR was performed in an ABI 9700 or Veriti thermocycler (Life Technologies, Carlsbad, CA) under the following cycling conditions: 5 min. initial denaturation at 95 °C:, followed by 40 cycles of 94 °C for 1 min., 62 °C for 30 s, and 72 °C for 1 min., with a final extension at 72 °C for 5 min. PCR product was then electrophoresed on a 1.5 % agarose gel containing SYBRsafe (Life Technologies). PCR products displaying a band in the expected ~500–600 base pair region were then purified by Exo-SAP-it (Affymetrix. Santa Clara, CA).

Molecular identification was done by Sanger sequencing of the 16S rDNA product with the same DPO primers used for PCR and BigDye Terminator v1.1 Cycle Sequencing Kit (Life Technologies) on an ABI Prism 3130×L sequencer (Life Technologies). A BLAST search against the IDNS Bacteria database (SmartGene IDNS, Lausanne, Switzerland) was done to provide a definitive identification of the organism to the species- or genus-level using the identity scores outlined by CLSI MM-18 [34].

### Ethics

This study was reviewed and approved by the Conjoint Health Research Ethics Board, University of Calgary (Ethics ID No: REB14-0588).

### Data analysis

Data was analyzed according to standard descriptive statistics. The sensitivity, specificity, positive and negative predictive values were calculated using the clinical diagnosis of IE based on the modified Duke criteria as the gold standard for diagnosis [3]. A true positive result was defined as one in which the valve culture or PCR sequencing was positive in a patient with a clinical diagnosis of IE. A false-positive result was one in which a microorganism was recovered from a patient without IE. A true negative culture was defined as a negative culture or PCR result in a patient without IE. A false-negative culture occurred when the PCR or culture was negative in a patient with IE.

## Results

### Patient characteristics

Sixty-eight patients were included in this study. Table 1 outlines key patient characteristics. Most of the patients were male (*n* = 52, 76.5 %), with mean age 53. One-third of the patients had a prosthetic valve (*n* = 23, 33.8 %). According to the modified Duke’s criteria, 50 patients (73.5 %) were diagnosed with definite IE and another 8 patients were classified as having possible IE. The remaining ten patients were controls and didn’t have IE.

**Table 1:** Clinical characteristics for all included patients

Demographics	Male (%)	52 (76.5 %)
	Age (years)	53.0 +/− 14.4
Medical history	Prosthetic valve	23 (33.8 %)
	Previous endocarditis	4 (6.0 %)
	Intravenous drug user	3 (4.4 %)
Laboratory data	White Blood Cell count (×10^9^/L)	13.8 +/− 6.4
	Peak ESR (mm/h)	58.5 +/− 31.7
	Peak CRP (mg/L)	118 +/− 94
	Transthoracic Echo Performed	53 (77.9 %)
	Trans-esophageal Echo Performed	63 (92.6 %)
Culture data	Mean sets of Blood Cultures	3.7 +/− 2.8
	Any positive blood culture	45 (66.2 %)
	Percentage of positive blood cultures	32.1 %
	Mean time from last positive blood culture until surgery (days)	15.4 +/− 24.7

Proportions are reported as number (percentage). Continuous variables are reported as number +/− standard deviation. *CRP* C-reactive protein, *ESR* erythrocyte sedimentation rate, *L* litre, *mg*/*L* milligram per litre, *mm*/*h* millimeters per hour

### Blood and heart valve culture

All patients had at least three sets of blood cultures drawn. Overall, any positive blood culture had a sensitivity of 77.6 % (45/58) and specificity of 100 % (10/10). In patients with negative blood cultures, PCR identified the microbiologic agent in 7/13 (54 %) patients. In patients with definite IE, any positive blood culture had a sensitivity of 88 % (44/50) and specificity of 94 % (17/18). In patients with definite IE and negative blood cultures, PCR identified the microbiologic agent in 5/6 (83 %) patients (*Staphylococcus lugdenensis*, *Streptococcus sanguinis*, *Streptococcus dysgalactiae*, *Propionibacterium acnes*, and *Escherichia coli*).

### Molecular testing of heart valve tissue

Table 2 shows the results of each patient’s blood culture, and heart valve tissue Gram stain, culture and 16S rDNA PCR result. Aortic valve replacement (40, 69 %) was more common in our patients than mitral valve surgery (18, 31 %). A total of 23 (33.8 %) patients had a prosthetic valve. The organisms causing IE in our patient cohort included: *Streptococcus viridans* group (21; 42 %), *Streptococcus agalactiae* (3; 6 %), *Enterococcus faecalis* (10; 20 %), *Staphylococcus* spp. (6; 12 %), *Haemophilus parainfluenzae* (2; 4 %), Gram-negative bacilli (2; 4 %), *Propionibacterium acnes* (1; 2 %), *and Cardiobacterium hominis* (1; 2 %). For patients with definite IE, broad-range 16S rDNA PCR/sequence agreed with blood or tissue culture results in 37 (74 %), and resulted in a new microbiologic diagnosis in 3 patients with identification of; *Streptococcus dysgalactiae*, *Propionibacterium. acnes*, *and Staphylococcus lugdenensis*. Additionally, 3 patients with possible IE but negative blood and heart valve tissue cultures had a positive PCR/sequencing results including; *Delftia tsuruhatensis*, *Streptococcus mitis*, *and Acinetobacter junii*. Clinical review of these 3 patients determined that they had IE, rather than these being false-positive PCR results. Finally, in one patient with a rejected diagnosis of IE, PCR sequencing suggested *Enterococcus faecalis*. In one patient with definite endocarditis, blood cultures suggested coagulase negative *Staphylococcus* but sequencing identified *Enterococcus faecalis*. This change would be expected to impact clinical management. Finally, in 13 patients with definite endocarditis the causative organism was clarified however the change would not be expected to impact the management plan. Overall, bacterial broad-range 16S rDNA PCR/sequencing contributed to the microbiologic diagnosis of 31 % of patients.

**Table 2:** Comparison of bacterial culture and ribosomal sequencing results

Duke’s criteria	Cardiac tissue	Tissue gram stain	Tissue culture	Blood culture	Fast DPO PCR	Bacterial 16S sequencing Identification	Diagnostic impact
Definite	MV	GPC	No growth	No growth	Positive	*Staphylococcus lugdenensis*	New
Definite	MV	Negative	No growth	No Growth	Positive	*Streptococcus dysgalactiae subsp. equisimilis*	New
Definite	PAV	GPB	Cons; broth only	No growth	Positive	*P. acnes*	New
Definite	MV	GPC	S. Mitis	No growth	Positive	*Streptococcus sanguinis*	Change
Definite	MV papillary leaflet	Negative	*S. bovis* group	*S. salivarius*	Positive	*S. gallolyticus subsp. pasteurianis*	Change
Definite	AV	GPC	No growth	*S. bovis*	Positive	*S. gallolyticus subsp. pasteurianis*	Change
Definite	MV Vegetation	Negative	No growth	*S. mitis*	Positive	*S. oralis*/*sanguinis*	Change
Definite	AV	Negative	No growth	*S. salivarius*	Positive	*S. mitis group* (*S. cristatus*/*oralis*/*mitis*)	Change
Definite	MV	Negative	No growth	*S. salivarius*	Positive	*S. mitis group* (*S. cristatus*/*oralis*/*infantis*)	Change
Definite	AV vegetation	Negative	*S. bovis* group	*S. bovis*	Positive	*S. gallolyticus subsp. gallolyticus*	Change
Definite	AV vegetation	Negative	No growth	GGS	Positive	*S. dysgalactiae subsp. equisimilis*	Change
Definite	PAV	Negative	*E. faecalis*/CONS; broth only	*S. agalactiae*	Positive	*S. agalactiae*	Change
Definite	AV vegetation	Negative	No growth	*S. mitis*	Positive	*S. sanguinis*	Change
Definite	PMV	GPC	*S. mitis* group	Group C Strep	Positive	*S. dysgalactiae subsp. equisimilis*	Change
Definite	AV	GPC	*S. mitis* group	*S. mitis*	Positive	*S. sanguinis*	Change
Definite	PAV	Negative	No growth	CoNS	Positive	*E. faecalis*	Change
Definite	MV	Negative	CoNS broth only	CoNS	Positive	*S. epidermidis*	Change
Definite	MV Vegetation	Mixed GPC	No growth	*S. mitis*	Positive	*S. mitis*	None
Definite	MV	Negative	*S. agalactiae*; broth only	*S. agalactiae*	Positive	*S. agalactiae*	None
Definite	AV abscess	Negative	No growth	*S. mitis*	Positive	*S. mitis group* (*mitis*/*tigurinus*)	None
Definite	AV abscess	Negative	*Enterococcus faecalis*; broth only	*E. faecalis*	Positive	*E. faecalis*	None
Definite	MV	Negative	No growth	*Haemophilus parainfluenzae*	Positive	*H. parainfluenzae*	None
Definite	AV	Negative	No growth	*Streptococcus pneumoniae*	Positive	*S. pneumoniae*	None
Definite	AV Vegetation	Positive	*E. faecalis*	*E. faecalis*	Positive	*E. faecalis*	None
Definite	MV	GPC	No growth	*S. mitis*	Positive	*S. mitis*	None
Definite	AV	Negative	*E. faecalis*	*E. faecalis*	Positive	*E. faecalis*	None
Definite	AV	Negative	No growth	*E. faecalis*	Positive	*E. faecalis*	None
Definite	AV	Negative	No growth	*E. faecalis*	Positive	*E. faecalis*	None
Definite	Aortic lesion	Negative	No growth	*Granulicatella adiacens*	Positive	*G. adiacens*	None
Definite	AV	GPC	No growth	*E. faecalis*	Positive	*E. faecalis*	None
Definite	AV	Negative	*S. lugdenensis*	*S. lugdenensis*	Positive	*S. lugdenensis*	None
Definite	AV	GPC	No growth	*S. mutans*	Positive	*S. mutans*	None
Definite	Aortic annulus	Negative	No growth	*S. mitis*	Positive	*S. mitis*	None
Definite	AV	Negative	*Cardiobacterium hominis*	*C. hominis*	Positive	*C. hominis*	None
Definite	AV leaflet	Negative	No growth	*S. agalactiae*	Positive	*S. agalactiae*	None
Definite	MV	Negative	*S. lugdenensis*	*S. lugdenensis*	Positive	*S. lugdenensis*	None
Definite	AV leaflet	Negative	*E. faecalis*	*E. faecalis*	Positive	*E. faecalis*	None
Definite	MV vegetation	Negative	*S. lugdenensis*; broth only	*S. lugdenensis*	Positive	*S. lugdenensis*	None
Definite	AV; left atrial lesion	Negative	*S. aureus*; broth only	*S. aureus*	Positive	*S. aureus*	None
Definite	AV	Negative	No growth	*S. mitis*	Positive	*S. mitis*/*sanguinis*	None
Definite	PAV	Negative	No growth	*S. salivarius*	Positive	*S. salivarius*	None
Definite	AV	Negative	*E. coli*	No growth	Positive	*E. coli*	None
Definite	MV vegetation	GPB	*H. parainfluenzae*	*H. parainfluenzae*	Positive	*H. parainfluenzae*	None
Definite	MV	Negative	CoNS; broth only	No growth	Negative	Not Done	None
Definite	MV	Negative	No growth	*E. faecalis*	Positive	*E. faecalis*	None
Definite	Aorta	Negative	No Growth	*E. faecalis*	Positive	*E. faecalis*	None
Definite	AV	Negative	No Growth	*S. aureus*	Negative	Not done	None
Definite	AV	Negative	No Growth	*S. aureus*	Positive	Mixed	None
Definite	AV vegetation	Negative	*S. bovis*	*S. bovis*	Positive	*S. bovis*	None
Definite	AV	Positive	*S. aureus*	*S. aureus*	Negative	Negative	None
Possible	PAV	Negative	No growth	No Growth	Positive	*Acinetobacter junii*	New
Possible	AV	Negative	No Growth	No Growth	Positive	*Delftia tsuruhatensis*	New
Possible	AV	Negative	No Growth	No Growth	Positive	*S. infantis*	New
Possible	AV	Negative	No growth	No growth	Negative	Negative	None
Possible	AV	Negative	No Growth	No Growth	Negative	Negative	None
Possible	PAV	Negative	No Growth	No Growth	Negative	Negative	None
Possible	AV	Negative	No Growth	No Growth	Negative	Negative	None
Possible	AV	Negative	No Growth	No Growth	Negative	Negative	None
Negative	AV	Negative	No Growth	No Growth	Positive	*E. faecalis*	New
Negative	MV	Negative	No Growth	No Growth	Negative	Negative	None
Negative	MV	Negative	No Growth	No Growth	Negative	Negative	None
Negative	PMV	Negative	No Growth	No Growth	Negative	Negative	None
Negative	AV	Negative	No Growth	No Growth	Negative	Negative	None
Negative	AV	Negative	No Growth	No Growth	Negative	Negative	None
Negative	AV vegetation	Negative	No Growth	No Growth	Positive	Mixed	None
Negative	MV thrombus	Negative	No Growth	No Growth	Negative	Negative	None
Negative	MV	Negative	No Growth	No Growth	Negative	Negative	None
Negative	AV	Negative	No Growth	No Growth	Positive	Mixed	None

*AV* aortic valve, *CoNS* coagulase negative *Staphylococcus*, *GBS* group B *Streptococcus*, *GCS* group C *Streptococcus*, *GGS* group G *Streptococcus*, *GPC* gram positive cocci, *GPB* gram positive bacilli, *MV* mitral valve, *PAV* prosthetic aortic valve, *PMV* prosthetic mitral valve. New indicates a new microbiologic diagnosis was made based on bacterial sequencing results. Change indicates that the microbiologic agent was changed or clarified

Table 3 shows the diagnostic performance of broad-range 16S rDNA PCR/sequencing compared to Gram stain and heart valve bacterial culture in patients with and without IE based on a definite diagnosis of IE. Molecular testing of heart valve tissue was positive in 46 (92 %) patients and negative or inconclusive in 4 (8 %) patients with definite IE. Most patients (*n* = 14; 78 %) who didn’t fulfill criteria for clinical IE had a negative ribosomal broad-range PCR result. Broad-range 16S rDNA PCR/sequencing had a sensitivity of 92 % (46/50) and specificity of 78 % (14/18). Tissue culture was positive in 23 patients and negative in 28, yielding a sensitivity of 46 % (23/50). Tissue gram stain was positive in 13 (26 %) of patients with confirmed IE.

**Table 3:** Performance of Gram stain and bacterial culture of heart valve tissue compared to 16S broad range PCR/sequencing for diagnosis of endocarditis

Method	Sensitivity	Specificity	PPV	NPV	Diagnostic efficiency
Gram Stain of Heart Valve Tissue	26.0 % (15.1–40.6)	100 % (78.1–100.0)	100 % (71.7–100.0)	32.7 % (21.0–46.8)	45.6 % (33.8–57.4)
Heart Valve Tissue Culture	46 % (33.0–59.6)	100.0 % (83.1–100.0)	100.0 % (83.1–100.0)	41.3 % (27.0–56.7)	60.2 % (50.8–68.0)
16S Broad Range PCR/Sequencing	92.0 % (84.6–96.5)	77.8 % (57.4–90.4)	92.0 % (84.6–96.5)	77.8 % (57.4–90.4)	88.2 % (77.4–94.9)

Diagnostic performance of tissue culture and 16S Broad Range PCR sequencing in 68 samples (i.e. 50 cases had Definite IE, 8 cases had Possible IE, and 10 Cases were controls with no clinical or laboratory evidence of IE). Calculations are based on a definite diagnosis of endocarditis by the Duke’s criteria as the gold standard for diagnosis. 95 % CI in brackets. *PPV* positive predictive value, *NPV* negative predictive value

When considering either definite or possible IE, broad-range 16S rDNA PCR/sequencing has a sensitivity of 84.4 % (49/58) and specificity of 90.0 % (9/10) compared to 37.9 % (22/58) and 100 % (10/10) for tissue culture respectively. Diagnostic accuracy for broad-range 16S rDNA PCR/sequencing was 85.3 % compared to 44.1 % for tissue culture. There were 17 patients with definite or possible prosthetic valve IE and PCR had sensitivity of 59 % (10/17) and specificity of 83 % (5/6), while tissue culture had respective values of 35 % (6/17) and specificity of 100 % (7/7).

### Effect of peri-operative antibiotic treatment

All patients with definite or possible IE were on appropriate antibiotics during the peri-operative period. On average, cardiac surgery was delayed an average of 15.4 days from the time of the patient’s last positive blood culture. Figure 1 shows that the prescription of antibiotic therapy had no effect on the bacterial isolate recovery from HV culture, or the ability of PCR/sequencing to detect the presence of bacterial DNA in excised heart valve tissue.

### Literature review

Table 4 summarizes the results for most of the previously published evaluation of user-developed broad-range 16S rDNA PCR/sequencing for patients with definite IE according to Duke’s criteria [9, 13–19, 23–28]. Only two studies that focused on patients with blood culture-negative endocarditis (BCNE) are included [29, 30]. In addition, there are two small evaluations of commercial molecular assays [31, 32]. A total of 824 patients with definite IE, 759 patients with blood culture-negative endocarditis, and 482 controls without IE have been studied using a molecular diagnostic approach, mainly in European centers. Although most of the patients in these studies were on antibiotics at the time of heart valve excision, only a few documented the median duration of pre-operative antibiotic treatment given prior to surgery. However, our patients’ experience was similar to those in other studies; at least two-weeks of antibiotics therapy had been given prior to cardiac surgery. Although some studies found that the duration of pre-operative antibiotics negatively effects heart valve culture results [18, 25], that has not been confirmed by others [9]. Pre-operative blood cultures are positive in most studies in approximately two-thirds of patients (range 33.3–87.7 %) which was confirmed in our patient population. However, heart valve culture had less optimal range of recovery of a pathogen (range 11.2–32.3 %) across these studies compared to either blood cultures or broad-range 16S rDNA PCR on heart valve tissue. User-developed broad-range 16S assays have also had a wide range of performance in patients with definite IE with a sensitivity (range = 41.2–100 %), specificity (range = 61.5–100 %). PPV (range = 79–100 %) and NPV (range = 34.4–100 %). Some of this variability may be explained by differences in gold standards for diagnosis. In studies where histology has also been reported, the highest correlation occurred between the presence of IE on histology and the HV PCR result [13, 15].

**Table 4:** Summary of heart valve 16S ribosomal PCR results from patients with definite IE according to Duke’s criteria: literature review

Study (Reference)	Location	Median duration of pre-operative antibiotic treatment (days)	Cases/Controls^a^	Cases with positive pre-operative blood culture(s)	Nucleotide position of primers in *E. coli* 16S rRNA gene	Agreement of HV culture and 16s PCR	HV culture sensitivity/specificity	Sensitivity	Specificity	PPV	NPV
1 (Goldenberger, [8])	Switzerland	NR ^b^	18	14/18 (77.8 %)	8-806	2/18 (11.1 %)	11.1/100 %	93.3 %	66.7 %	93.3 %	66.7 %
2 (Gauduchon, [13])	France	31.5 (range, 8 to 150)	29/23 ^c^	21/29 (72.4 %)	911-930 and 1390-1371	27/29 (93.1 %) ^c^	NR	ND	ND	ND	ND
3 (Lang, [19])	UK	NR ^d^	28/61	20/28 (71.4 %)	1522-1540 and 1170-1189	14/20 (70 %)	NR	ND	ND	ND	ND
4 (Breitkopf, [23])	Germany	NR	51/16	7/21 (33.3 %)	8-27 and 907-926	None	7.8/93.7 %	41.2 %	100 %	100 %	34.8 %
5 (Greub, [15])	France	NR	127/118	57/127 (44.9 %)	536-1050	14/68 (20.6 %)	13/98 %	61 %	100 %	100 %	74 %%
6 (Rovery, [24])	France	19.5 (range, 1–150)	147	NR	536-1050	64/95 (67 %) ^e^	NR	ND	ND	ND	ND
7 (Kotilainen, [18])	Finland	19.6 (range 1–58d)	28/18	20/28 (71.4 %)	1054-1077 and 1950-1926	18/25 (72 %)	13.1/100 %	43.1 %	100 %	100 %	58.1 %
8 (Marin, [9])	Spain	10 (range, 1–25)	35/120	31/35 (88.6 %)	783-806 and 1389-1370	16/35 (45.7 %)	24.3/56.4 %	96 %	95.3 %	98.4 %	88.5 %
9 (Volstedlund, [25])	Denmark	19.3 (range, 0–90)	57/10	50/57 (87.7 %)	341-534	19/57 (33.3 %)	26/62 %	72 %	100 %	100 %	62 %
10 (Fournier, [30]) ^g^	France	NR	549/191/19	All patients had negative blood culture	Same as study (2) 536F and RP2	45.6 %	45.7 %/NR	69.2 %	ND	ND	ND
11 (Miyazato, 2011)	Japan		19	15/19 (79 %)	8UA and 1485B	None: 79 % had a positive Gram stain	None were positive	100 %	100 %	79 %	100 %
12 (Vondracek, [27])	Sweden	NR	57/61	48/57 (84 %)	334-939	20/57 (35.1 %)	23/87 %	77 %	100 %	100 %	87 %
13 (Boussier, [28]) ^f^	France	NR	31	23/31 (74.2 %)	Same as (2) and 8-27 and 1510-1492	5/31 (16.1 %)	32.3/100 %	78 %	100 %	100 %	61.5 %
14 (Kemp, [17])	Denmark	NR	56/36	36/56 (64.3 %)	8-534	7/42 (16.7 %)	16.7/100 %	88 %	61.5 %	88 %	57.1 %
15 (Sadaka, [29]) ^g^	Egypt	NR	19	All blood cultures were negative	1522-1540 and 1170-1189	5/6 (83.3 %)	62.5 %/NR	ND	ND	ND	ND
16 (Harris, [16])	Ireland, UK	NR	47	35/47 (74.5 %)	16S Fa, 16S Fb and 16SR (320 bp)	29/47 (61.7 %)	NR	67 %	91 %	96 %	46 %
17 (Leli, [31])^h^	Italy	NR	20	NR	Septifast (Roche)	3/19 (15.8 %)	15.8/100 %	95 %	100 %	100 %	83.3 %
18 (Marsch, [32]) ^i^	Germany	NR	46	NR	UMD™, Molzym	27/46 (56.7 %)	32.1/100 %	61 %	ND	ND	ND

^a^Only data for definite IE cases is included. Some studies (3, 7, and 9) also enrolled a small number of cases with possible IE according to Duke’s criteria. Controls were patients undergoing cardiac surgery for non-infective reasons

^b^Not reported (NR)

^c^Definite IE was diagnosed by histology in the 52 cases; 29 definite IE cases were included and 23 cases with no evidence of IE on histology of heart valve tissue. PCR results were compared to histology and not culture in this study

^d^Cases divided into an active group (*n* = 19) that was on antimicrobial therapy at the time of surgery, and the resolved group (*N* = 9) who had completed treatment (time period 3 months to 7 years after treatment at the time of cardiac surgery, and nine others with possible IE

^e^Bacterial DNA was amplified by PCR significantly more often (64/95, 67 %) in HV with histological evidence of IE than valves that had no histological evidence of IE (21/55, 38 %) (*p* = 0.001)

^f^This study used a two-step PCR procedure: first, a real-time method, then a conventional end-point PCR was applied to HV samples to improve the sensitivity of molecular detection from 38 to 58 %

^g^Both of these studies only enrolled patients with culture negative endocarditis (i.e., all patients had clinical evidence of IE but negative blood cultures)

^h^PCR on heart valve tissue was performed using a commercial real-time PCR assay (SeptiFast, Roche Molecular Systems, Mannheim Germany)

^i^PCR of heart valve tissue was performed using a commercial assay (UDM™, Molyzm, Bremen, Germany) that uses both 16S rRNA bacterial primers and 18S rRNA fungal primers

A wide range of primers that have different nucleotide positions in the *E. coli* 16S rRNA gene have been used in user-developed assays, which is part, may explain the wide range in performance in previously reported studies (Table 4). However, both commercial assays also showed similar performance variability (i.e., sensitivity 61 vs. 95 %) albeit the Septifast (Roche) and UDM™ (Molyzym) assays have different configurations, and have only been evaluated in very small numbers of patients [31, 32].

## Discussion

This is the first evaluation of a novel fast broad-range 16S rDNA PCR/sequencing assay in a Canadian patient population, and the first study of the clinical utility of DPO primers for the routine molecular analysis of heart valve tissue in consecutive patients with and without infective IE. Additionally, our study had the advantage of extensive clinical information to better delineate the Dukes minor criteria for all patients. Our study confirms the higher sensitivity of molecular heart valve testing compared to tissue culture [35–38]. However, our novel assay has one of the highest reported sensitivities of a user-developed broad-range 16S rDNA PCR to date. Although pre-operative blood cultures make a microbiologic diagnosis in approximately two-thirds of patients suspected of having IE, subsequent molecular analysis of heart valve tissue contributed to the microbiologic diagnosis of 31 % of our patients. However, the change in diagnosis would only be expected to contribute to the clinical management of 13 % of patients. There was a significant delay between first culture results and the time of surgery, during which patients were universally on antibiotics. This is known to interfere with culture results but did not seem to significantly impair the diagnostic utility of molecular sequencing [39]. Broad-range 16S rDNA PCR/sequencing seems particularly useful in patients with culture-negative IE, which has also been reported recently by another center [38]. Additionally, sequencing to the subspecies level, as we have done, has important clinical implications in a small but important subset of patients [40]. Therefore, bacterial broad-range 16S rDNA PCR/sequencing has a complementary role with traditional culture techniques in the diagnosis of potential IE.

In our study the specificity of broad-range 16S rDNA PCR/sequencing was relatively lower than previous reports, despite the use of DPO primers that have been shown to substantially decrease cross-reactivity with human DNA [35–38]. This may reflect occult infection in patients who otherwise did not meet the Duke criteria for infective endocarditis. This further supports the suggestion of the addition of molecular diagnostics to the Duke criteria [12]. Alternatively, this could reflect sample contamination at the time of collection prior to sequencing, or within one of the molecular test components. Until the clinical role for broad-range 16S rDNA PCR/sequencing is clarified positive PCR results in patients who otherwise do not meet criteria for IE should therefore be interpreted within the full clinical context and correlated with all other laboratory results.

Prosthetic valve IE accounts for up to 20 % of cases of IE and is associated with high rates of morbidity and mortality [41], making microbiologic diagnosis especially critical. As implantation of prosthetic valves increases, this group of patients will become increasingly important [42]. Previous investigations looking at PCR sequencing in patients with mechanical or bio-prosthetic valves showed poor diagnostic performance [5, 36]. Given the limited specimen available from prosthetic valves, sampling error is an important source of diagnostic error. Sequence amplification requires smaller samples, making sequencing potentially less prone to sampling errors than bacterial culture or histologic analysis [5]. In our study, the sensitivity of broad-range 16S rDNA PCR/sequencing in prosthetic valves was lower compared to its performance in native valves but still outperformed tissue culture.

Our study has several important limitations. We are reporting the experience from a single centralized laboratory that has accrued experience in the development and implementation of complex user designed molecular assays. The results reported herein may not reflect the accuracy of the utilization of this technology in jurisdictions without expertise in broad-range 16S rDNA PCR/sequencing. Additionally, our case series is limited by selection bias, since only patients undergoing valve surgery were included in our study. While this limits the generalizability of our study, it accurately reflects routine sequencing of excised heart valve tissue. Finally, the Duke criteria that we used as the gold standard for diagnosis are reliant on tissue and blood culture as diagnostic criteria, which may lead to overestimates of their diagnostic performance.

## Conclusion

Identification of the underlying microbial pathogen in endocarditis is crucial to determining length of therapy and appropriately targeting anti-microbial. Bacterial sequencing to the subspecies level improves pathogen identification, and has important clinical implications. Broad range sequencing performed well as a diagnostic test, contributing the clinical care of 13 % of patients, compared to a gold standard of clinical diagnosis based on Dukes criteria. This procedure should be considered an essential step in the management of all patients with suspected or possible infective endocarditis, particularly in patients with culture negative IE.
